# Supplementary material for: Hemoadsorption Therapy for Critically Ill Patients with Acute Liver Dysfunction: A Meta-Analysis and Systematic Review
Source: Biomedicines. 2023 Dec 27;12(1):67. doi: 10.3390/biomedicines12010067 (PMC10813081; doi:10.3390/biomedicines12010067)
Supplement: Supplementary file 1 [file biomedicines-12-00067-s001.zip › Table S1 - GRADE.pdf]

Hemoadsorption therapy compared to no treatment for critically ill with acute liver dysfunction

Patient or population: critically ill with acute liver dysfunction

Setting:

Intervention: hemoadsorption therapy

Comparison: no treatment

Outcomes	№ of participants (studies) Follow-up	Certainty of the evidence (GRADE)	Relative effect (95% CI)	Anticipated absolute effects	
				Risk with no treatment	Risk difference with hemoadsorption therapy
Total bilirubin (pooled)	4 (4 observational studies)	⊕⊕◯◯ Low	-	-	MD **4.79 mg/dL lower** (6.25 lower to 3.33 lower)
Creatinine (pooled)	3 (3 observational studies)	⊕⊕◯◯ Low	-	-	MD **0.38 mg/dL lower** (1.27 lower to 0.5 higher)
ALT (individual)	7 (7 observational studies)	⊕⊕◯◯ Low	-	-	**0** (0 to 0)
AST (individual)	8 (8 observational studies)	⊕⊕◯◯ Low	-	-	**0** (0 to 0)
Creatinine (individual)	6 (6 observational studies)	⊕⊕◯◯ Low	-	-	**0** (0 to 0)
CRP (individual)	7 (7 observational studies)	⊕⊕◯◯ Low	-	-	**0** (0 to 0)
Total bilirubin (individual)	(7 observational studies)	⊕⊕◯◯ Low	-	-	**0** (0 to 0)

Hemoadsorption therapy compared to no treatment for critically ill with acute liver dysfunction

Patient or population: critically ill with acute liver dysfunction

Setting:

Intervention: hemoadsorption therapy

Comparison: no treatment

Outcomes	№ of participants (studies) Follow-up	Certainty of the evidence (GRADE)	Relative effect (95% CI)	Anticipated absolute effects	
				Risk with no treatment	Risk difference with hemoadsorption therapy
Vasopressor need (individual)	7 (7 observational studies)	⊕⊕◯◯ Low	-	-	**0** (0 to 0)

***The risk in the intervention group** (and its 95% confidence interval) is based on the assumed risk in the comparison group and the **relative effect** of the intervention (and its 95% CI).

CI: confidence interval; **MD:** mean difference

GRADE Working Group grades of evidence
High certainty: we are very confident that the true effect lies close to that of the estimate of the effect.
Moderate certainty: we are moderately confident in the effect estimate: the true effect is likely to be close to the estimate of the effect, but there is a possibility that it is substantially different.
Low certainty: our confidence in the effect estimate is limited: the true effect may be substantially different from the estimate of the effect.
Very low certainty: we have very little confidence in the effect estimate: the true effect is likely to be substantially different from the estimate of effect.
