# Supplementary figures and images for: Hemoadsorption Therapy for Critically Ill Patients with Acute Liver Dysfunction: A Meta-Analysis and Systematic Review
Source: Biomedicines. 2023 Dec 27;12(1):67. doi: 10.3390/biomedicines12010067 (PMC10813081; doi:10.3390/biomedicines12010067)

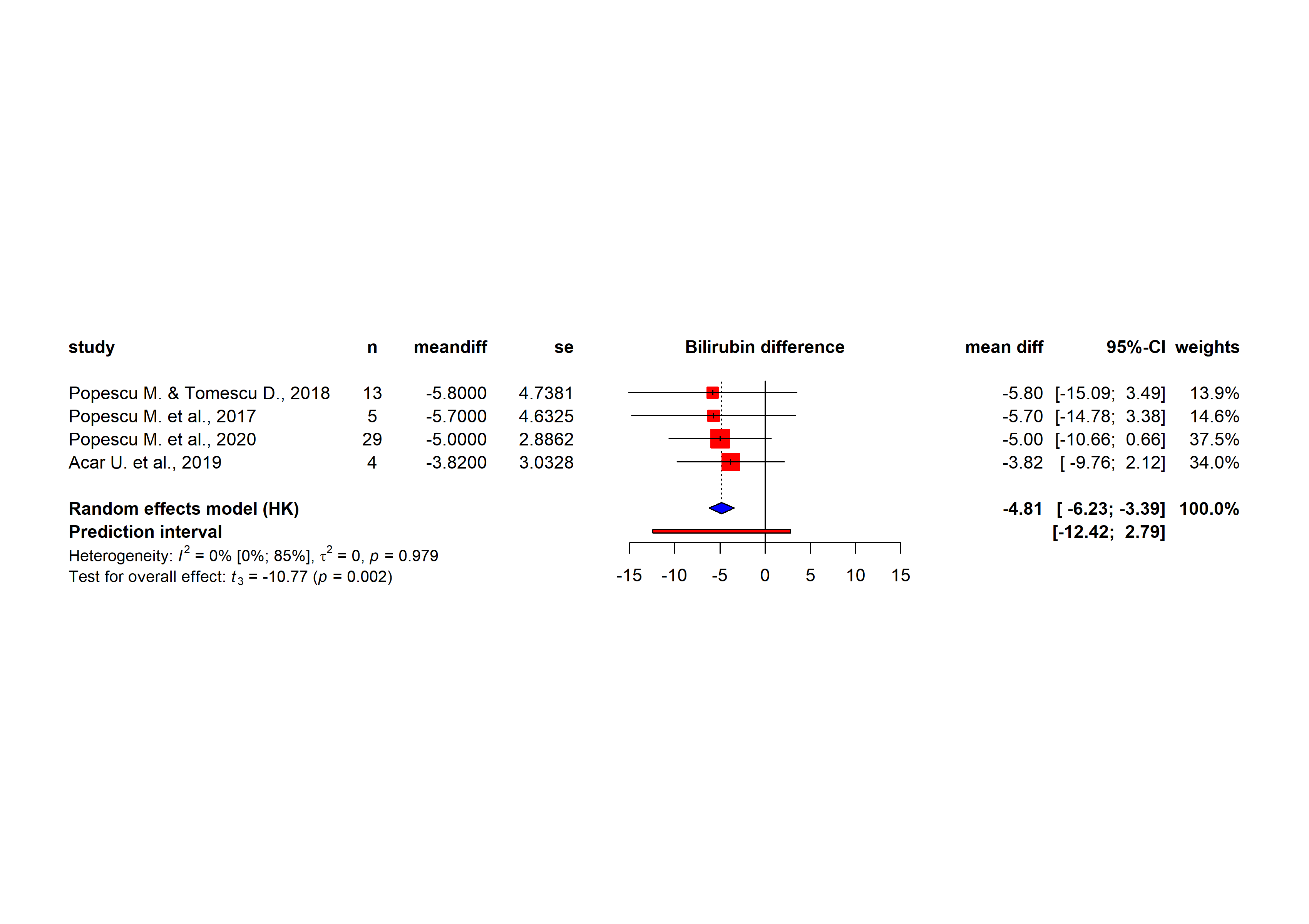

Supplement: Supplementary file 1 [file biomedicines-12-00067-s001.zip › Figure S1 - zero correlation bilirubin.png]

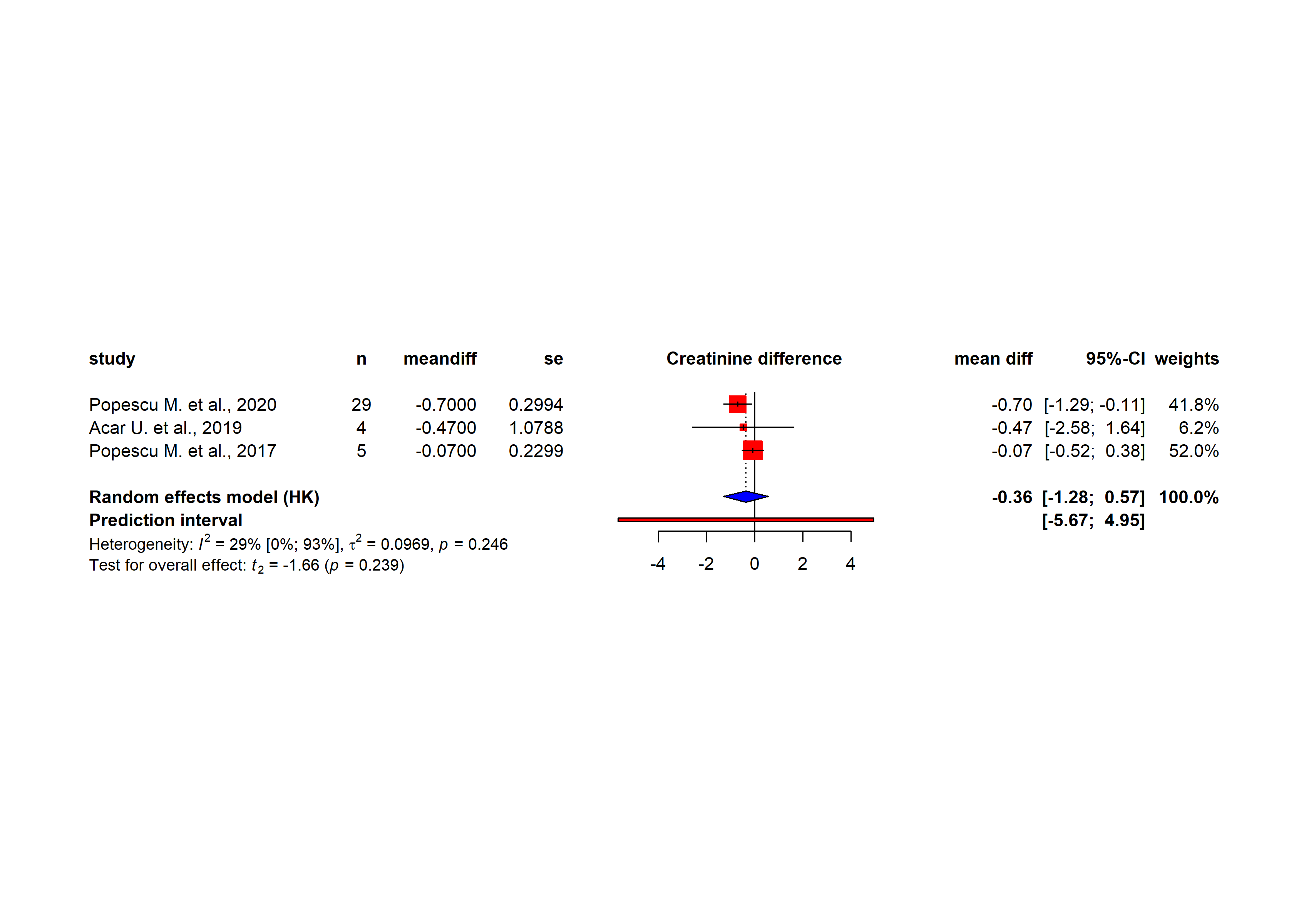

Supplement: Supplementary file 1 [file biomedicines-12-00067-s001.zip › Figure S2 - zero correlation creatinine.png]

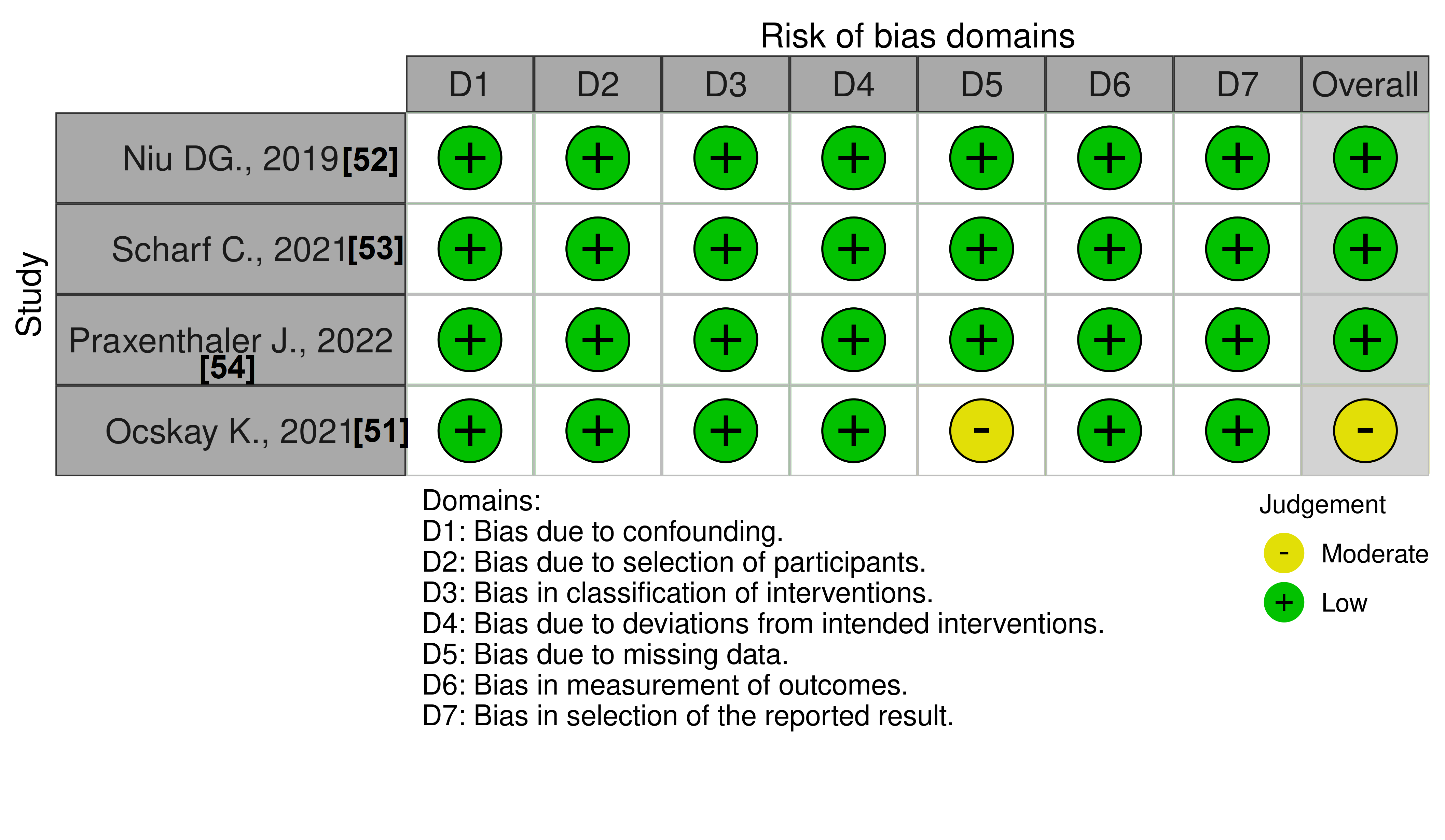

Supplement: Supplementary file 1 [file biomedicines-12-00067-s001.zip › Figure S3 - ROBINS-I.png]

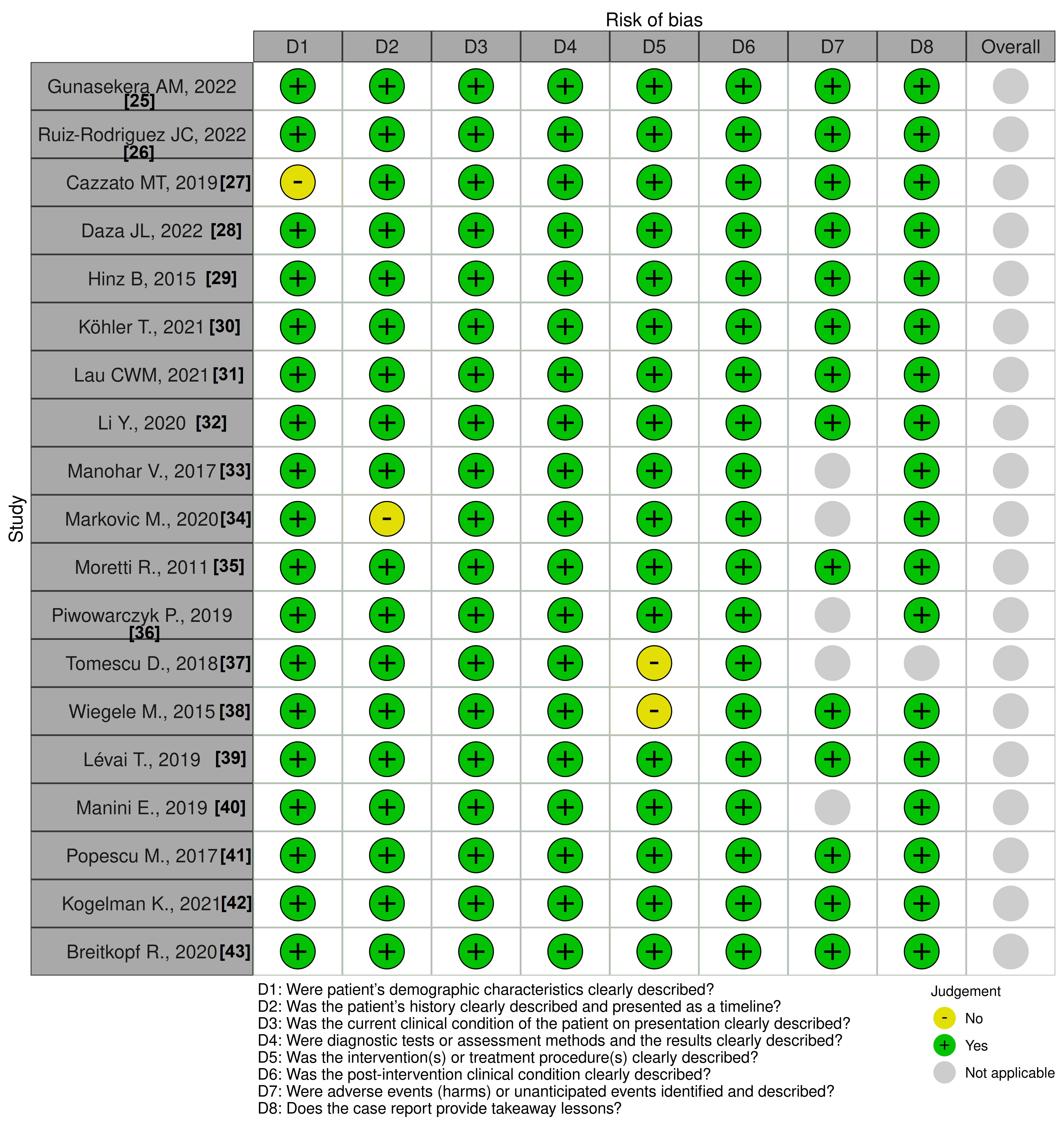

Supplement: Supplementary file 1 [file biomedicines-12-00067-s001.zip › Figure S4 - JBI Case reports.png]

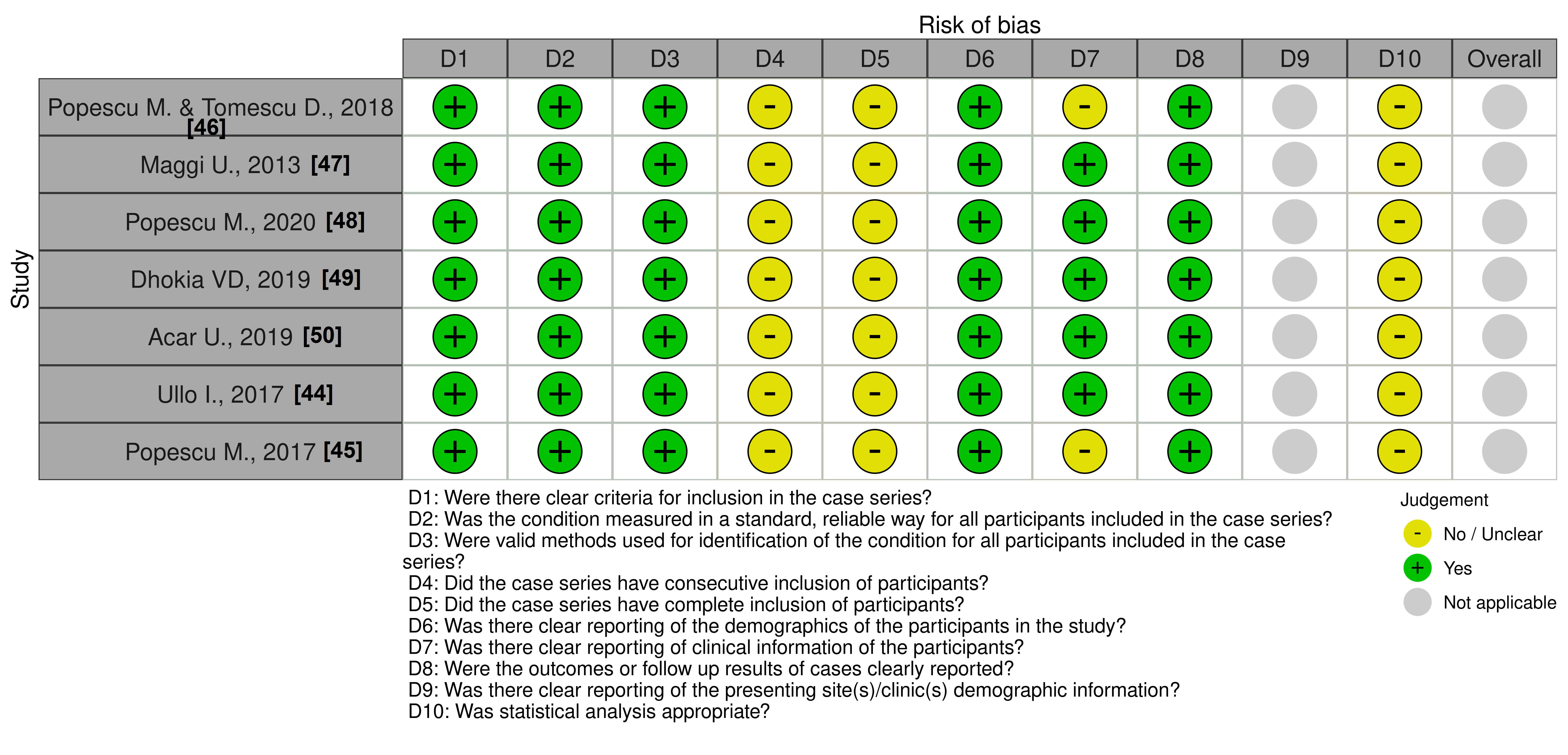

Supplement: Supplementary file 1 [file biomedicines-12-00067-s001.zip › Figure S5 - JBI Case series.png]
